# Association between Iodine Nutrition Status and Thyroid Disease-Related Hormone in Korean Adults: Korean National Health and Nutrition Examination Survey VI (2013–2015)

**DOI:** 10.3390/nu11112757

**Published:** 2019-11-13

**Authors:** Sohye Kim, Yong Seok Kwon, Ju Young Kim, Kyung Hee Hong, Yoo Kyoung Park

**Affiliations:** 1 Department of Medical Nutrition, Graduate School of East-West Medical Science, Kyung Hee University, Yongin 17104, Korea; sohye76@daum.net; 2 Nutrition Care Services, Seoul National University of Bundang Hospital, Seongnam 13620, Korea; 3 F&D Communication, Gyeonggi 10433, Korea; shafrang@naver.com; 4 Department of Family Medicine, Seoul National University of Bundang Hospital, Seongnam 13620, Korea; kkamburi@gmail.com; 5 Department of Food Science and Nutrition, Dongseo University, Pusan 47011, Korea; hkhee@gdsu.dongseo.ac.kr

**Keywords:** iodine nutrition status, thyroid disease, thyrotropin, urine iodine, epidemiologic studies, Korean

## Abstract

This study aimed to observe the relationship between iodine nutrition status (dietary iodine intake and estimated iodine intake based on urinary iodine concentration (UIC)) and thyroid disease-related hormones. This study involved 6090 subjects >19 years old with valid UIC, assessed between 2013 and 2015 by the Korean National Health and Nutrition Examination Survey, using a stratified, multistage, clustered probability-sampling design. The estimated iodine intake in participants was measured using UIC and urine creatinine. To examine the effect of iodine intake on thyroid disease, the iodine intake was divided into Korean Dietary Reference Intakes groups, and logistic regression analysis was performed via the surveylogistic procedure to obtain odds ratios (ORs) and 95% confidence intervals (CIs). The estimated iodine intake showed a significant positive correlation with dietary iodine intake (r = 0.021, *p* < 0.001), UIC (r = 0.918, *p* < 0.001), and thyroid-stimulating hormone (TSH) (r = 0.043, *p* < 0.001), but a significant negative correlation with free thyroxine (FT4) (r = −0.037, *p* < 0.001). Additionally, as the estimated iodine intake increased, age, TSH, and UIC increased, but FT4 decreased (*p* for trend < 0.0001). The risk of thyroid disease was higher in the “≥tolerable upper intake level (UL ≥ 2400 µg/day)” group than in the “<estimated average requirement (EAR < 150 µg/day)” group in females (OR: 2.418; 95% CI: 1.010–5.787). Also, as iodine intake increased, the risk of thyroid disease increased (*p* for trend < 0.038).

## 1. Introduction 

Korea is geographically rich in iodine and one of the iodine-rich countries with a high intake of seaweeds [1]. According to a recent study, the mean dietary iodine intake was 763.5 μg for Korean female subjects and 953.1 μg for males [2]. In a study involving the trend analysis of iodine intake, the iodine intake for males was 326.2–817.0 µg and for females 257.0–802.4 μg [3]. It seems that a majority of the studies indicate that most of the Koreans’ iodine intake is within the upper limit (UL) (2400 μg) intake, but over two times higher than the recommended nutrient intake (RNI) (150 μg) [4,5,6,7,8]. Iodine controls the speed of, and is therefore an essential element in, thyroid hormone synthesis. When the iodine intake is insufficient, the hypothalamus hormones, thyrotropin-releasing hormone (TRH) is secreted, and thyroid-stimulating hormone (TSH) further increases the secretion of the thyroid to increase the synthesis and secretion of the thyroid hormone. When blood levels of triiodothyronine (T3) and thyroxine (T4) decrease, and in response the thyroid activity is increased to compensate for iodine deficiency, a sudden excessive intake can increase the risk of hyperthyroidism. Additionally, excessive iodine intake can change the thyroid function, and according to several studies, the prevalence of hypothyroidism, hyperthyroidism, and autoimmune thyroiditis has increased [9,10,11,12,13]. Many studies have reported that deficiency and excess of iodine are associated with thyroid dysfunction [7,10,14,15]. Therefore, it is important to find out effects on thyroid hormones made by level of iodine nutrition status. The iodine nutrition status of the population is measured based on dietary iodine intake or urinary iodine concentration (UIC), which is a well-accepted, cost-effective, and readily available indicator. Furthermore, according to the iodine nutritional epidemiology standard of the World Health Organization (WHO), UIC is a recommended barometer for iodine intake that assesses the iodine status of the population and is a sensitive indicator [16,17].

TSH, T4, T3, and thyroid autoantibodies (TPOAb) measurements are biochemical tests in the diagnosis and control of thyroid disease [18]. Serum TSH is the most sensitive marker for assessing the status of thyroid function and measurement of serum TSH level is used as a screening test for patients with thyroid dysfunction [19]. TSH is known to be affected by factors such as age, sex, race, iodine intake, smoking, presence of antibodies, and body mass index (BMI) [15,20].

The purpose of this study is to investigate the relationship between iodine nutrition status (dietary iodine intake and estimated iodine intake based on UIC) and thyroid disease-related hormones, such as serum TSH and free thyroxine (FT4). Also, we investigated the association of the iodine nutrition status and the thyroid disease incidence among Korean adults.

This is the nationwide study to observe the relationship between thyroid disease-related functions, such as serum TSH and FT4 level, and UIC, which was first introduced by the Korean National Health and Nutrition Examination Survey (KNHANES) VI (2013–2015), and also examined the relationship between iodine nutrition status including iodine intake in people’s diet and estimated iodine intake.

## 2. Methods and Materials

### 2.1. Study Population

The KNHANES is conducted to obtain national estimates of the health and nutrition status of Koreans by the Korea Centers for Disease Control and Prevention (KCDC) that uses a stratified, multistage clustered probability-sampling design [21]. KNHANES is a nationwide, population-based, cross-sectional study to assess the health and nutrition status of the Korean civilian, noninstitutionalized population. Each survey consists of three sections: health interview, health examinations, and nutritional survey.

We selected from the total population (*n* = 22,938) after the exclusion of <19 years (*n* = 4914), subjects with thyroid cancer (*n* =135), pregnant and lactating women (*n* = 239), subjects reporting unrealistic daily total energy intakes (<500 kcal, >5000 kcal) (*n* = 2251), subjects who did not test UIC (*n* = 9312), and subjects with missing weight variables (*n* = 2). As a result, a total of 6095 subjects were included in the final analysis (men=2852, women=3243). This subsample of KNHANES VI (2013–2015) consisted of 6095 participants who underwent the thyroid function test (serum TSH and FT4) and UIC stratified subsampling with consideration of sex and age.

This study protocol was approved by the Institutional Review Board of the KCDC and the KNHANES (2013-07CON-03-4C, 2013-12EXP-03-5C, and 2015-01-02-6C). All participants gave written informed consent.

### 2.2. Measurement of TSH, FT4, and UIC

Serum TSH and FT4 levels analyzed via electrochemiluminescence immunoassay were used. Serum TSH (reference range 0.35–5.50 mU/L) and FT4 (reference range 0.89–1.76 ng/dL) levels were analyzed using E-TSH kit (Roche Diagnostics, Mannheim, Germany) and E-Free T4 kit (Roche Diagnostics, Mannheim, Germany), respectively. UIC, analyzed through inductively coupled plasma mass spectrometry (ICPMS; PerkinElmer, Waltham, MA, USA) using iodine standard (Inorganic Venture, Christiansburg, VA, USA), was used [22].

### 2.3. Estimated Iodine Intake

The estimated iodine intake in populations was calculated by measuring the UIC and urine creatinine (Ucr) level and the following equation (1) [6,23,24]:

Estimated of iodine intake (µg/day) = UIC (µg/L) × {879.89 + (weight (kg) × 12.51) − [(6.19 × age) + (34.51 if black) − (379.42 if female)]} / (Ucr × 0.92 × 10).(1)

### 2.4. Establishment of Iodine Database of Commercial Foods in Korea

The iodine content of foods was based on the values shown in the Food Values of the Korean Nutrition Society [25], Food Composition Tables, 9th revision by the Korean National Institute of Agricultural Science, Rural Development Administration [26], a thesis that established the iodine database for common Korean foods [2], and the Standard tables of food composition in Japan (7th revised version) of the Ministry of Education, Culture, Sport, Science, and Technology, Tokyo, 2015 [27].

The value was selected if the food source existed on the database; however, if there was no matching food, it was replaced by something a similar food item from the database. If there were variations in terms of the processing method for certain foods, such as drying methods, we calculated the iodine values based on the values of the existing source of the foods. Moreover, if there were multiple values from different sources of data for one specific food, the mean value for the specific food was used. The total number of food items was 837, and the number of foods with iodine content was 559, which provided 66.8% coverage.

### 2.5. Measurement of Dietary Iodine Intake Using 24-hr-Dietary Recall

The nutrition survey data were collected using the 24-h dietary recall method and face-to-face health interviews by trained dietitians and health examination [28]. The daily intake of energy was calculated using the Korean Foods and Nutrients Database of the Rural Development Administration [26]. To calculate the dietary iodine intake, we established an iodine database by merging the data on food items from the 24 h diet recall in the KNHANES database (2013–2015) with the established iodine value for each food item.

### 2.6. Statistics

As the KNHANES data is based on stratified multistage probability extraction rather than simple random extraction data, this study analyzed the weight (2013, KNHANES; Wt_hmnt, 2014, 2015 KNHANES; Wt_trnt), the stratification variable (KSTRATA) and the primary sampling unit (PSU). To test for significant differences, the *t*-test was used when calibration was not performed using the surveyreg procedure, and the general linear model was used when calibration was performed using the surveyreg procedure. Additionally, the age and total daily energy intake were used as calibration variables. In addition, the correlation between each of the iodine intake and thyroid function tests was analyzed using Pearson correlation. Using the paired *t*-test, associations were determined with weighted measures among the estimated iodine intake, dietary iodine intake, and UIC. Lastly, to examine the effect of iodine intake on thyroid disease, this study divided iodine intake into Korean Dietary Reference Intakes (KDRI) groups [4], and logistic regression analysis was performed via the surveylogistic procedure to obtain odds ratios (OR) and 95% confidence intervals (CI). All statistical analyses were performed using SAS Ver. 9.4 (SAS Institute, Cary, NC, USA).

## 3. Results

### 3.1. Iodine Nutrition Status

**Figure 1:**
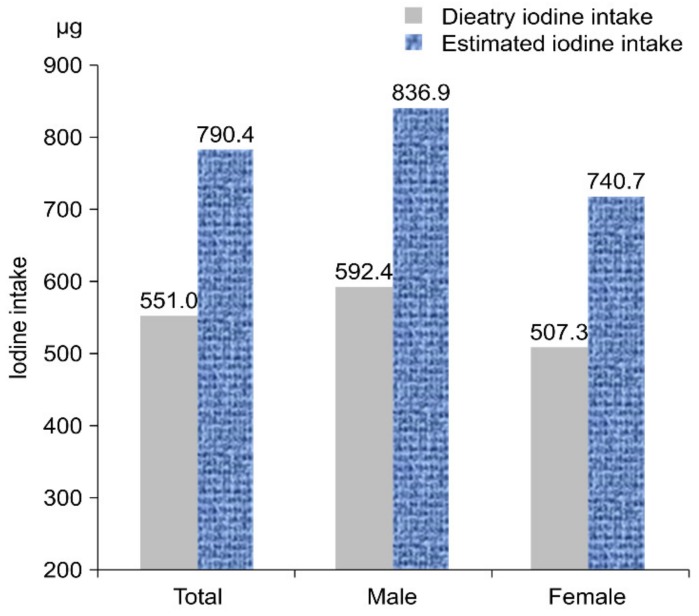
Mean of dietary iodine intake vs. estimated iodine intake.

The mean estimated iodine intake for all subjects was 790.1 µg, which was higher than the mean dietary iodine intake (551.0 µg). These results were identical for both the male and female groups (Figure 1).

The results of the analysis of the iodine nutrition status of the subjects are shown in Table 1. The thyroid disease group was higher in the total subjects’ energy than in the non-thyroid disease group (unadjusted *p* < 0.0001, adjusted for age *p* = 0.001). In total subjects, the mean dietary iodine intake of the non-thyroid disease group was 554.0 ± 35.8 μg, which was higher than that of the thyroid disease group of 458.8 ± 93.6 μg. However, the mean of estimated iodine intake of the non-thyroid disease group was 780.0 ± 56.1 μg, which was lower than that of the thyroid disease group of 1108.1 ± 195.8 μg. In total subjects, the mean UIC of the non-thyroid disease group was 883.2 ± 92.1 μg, which was lower than that of the thyroid disease group of 1085.9 ± 183.9 μg, but there was no significant difference. Additionally, the same result was obtained in the female group (non-thyroid disease 849.8 μg, thyroid disease group 1145.5 μg), but the result of the male group was the opposite (non-thyroid disease 913.5 μg, thyroid disease group 745.1 μg), and there were no significant differences between these groups. Moreover, there were no significant differences in all groups with UIC, dietary iodine intake, and estimated iodine intake.

### 3.2. TSH and FT4 of the Subjects

**Figure 2:**
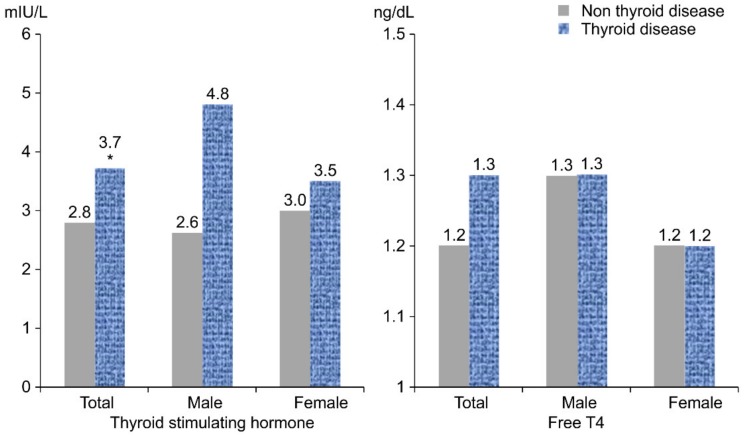
TSH and FT4 of thyroid disease status by sex.

The TSH and FT4 levels of the non-thyroid disease group vs. the thyroid disease group are shown in Figure 2. The mean TSH of the non-thyroid disease group was 2.8 ± 0.1 mIU/L, which was lower than that of the thyroid disease group (3.7 ± 0.4 mIU/L) (adjusted for age and energy intake *p* = 0.029). As a result, TSH in the total subjects was significantly higher in the thyroid disease group than in the non-thyroid disease group. Male and female groups showed similar results, but there were no significant differences. Additionally, the mean FT4 of the non-thyroid disease group was lower than that of the thyroid disease group, but there was no significant difference.

### 3.3. UIC, TSH, and FT4 According to KDRI of the Estimated Iodine Intake

The estimated iodine intake was divided into KDRI groups, and the results of UIC, TSH, and FT4 tests for each group are presented in Table 2.

**Figure 3:**
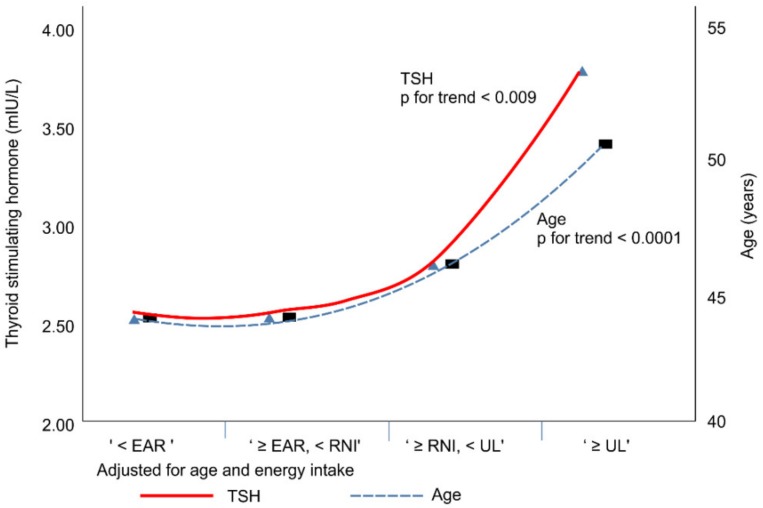
Correlation of TSH and age with the estimated iodine intake.

**Figure 4:**
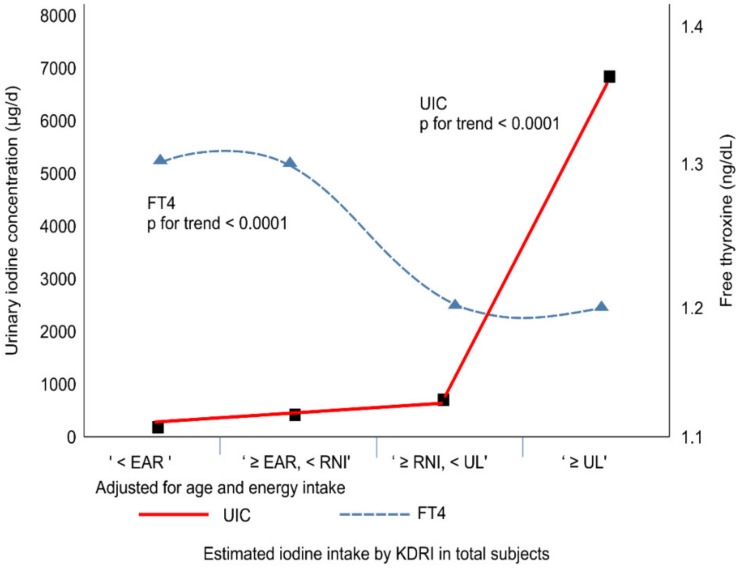
Correlation of UIC and free thyroxine (FT4) with the estimated iodine intake.

The KDRI groups were as follows: “<EAR (estimated average requirement)”, <95 µg/day; “≥EAR, <RNI (recommended nutrient intake),” ≥95 µg/day, <150 µg/day; “≥RNI, <UL (tolerable upper intake),” ≥150 µg/day, 2400 µg/day; and “≥UL,” ≥2400 µg/day by KDRI [4]. The mean and median values of the UL group were dramatically higher than those of the other groups (Figure 3). In total subjects, both unadjusted and adjusted for age and energy intake, as iodine intake increased, age (*p* for trend < 0.0001), TSH (*p* for trend = 0.009) (Figure 3), and UIC (*p* for trend < 0.0001) tended to increase, but FT4 showed a tendency to decrease (*p* for trend < 0.0001) (Figure 4). In addition, in both male and female groups, as iodine intake increased, age, TSH, and UIC tended to increase, but FT4 showed a tendency to decrease. All results are significant except for TSH in the female group.

### 3.4. Prevalence of Thyroid Disease and Distribution of Iodine Intake According to the Estimated Iodine Intake by KDRI

The intake range, median, and mean of the estimated iodine intake classified by KDRI groups are shown in Table 3.

**Figure 5:**
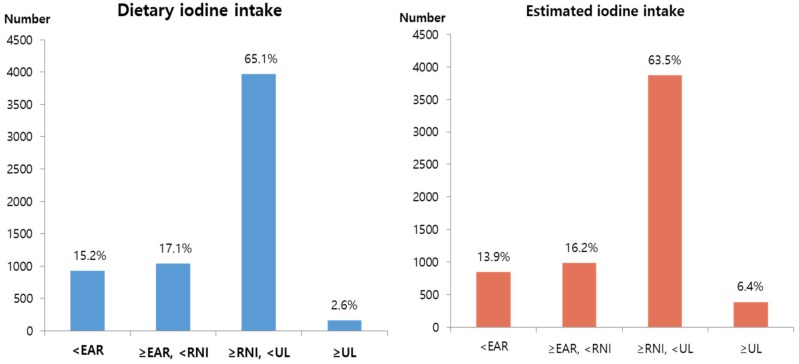
Population distribution of iodine intake by KDRI in the total subjects.

The population distribution with respect to the dietary iodine intake and estimated iodine intake of all the subjects was divided into KDRI groups. The “≥RNI, <UL” group had the largest population—65.1% with respect to dietary iodine intake and 63.5% with respect to estimated iodine intake, while the “UL” group had the smallest population (Figure 5).

### 3.5. Relation between Thyroid Disease and the Estimated Iodine Intake by KDRI

The results of the relationship between the estimated iodine intake and the risk of thyroid disease incidence according to KDRI groups using logistic regression analysis are presented in Table 4.

In relation to the thyroid disease according to the estimated iodine intake, as the estimated iodine intake increased in Model 1, which was the unadjusted model in the female group, the odds ratio of the “≥UL” group was 2.940 (95% CI: 1.267–6.823), which showed a tendency to increase the risk of thyroid disease incidence (*p* for trend = 0.014). Additionally, as the iodine intake increased in Model 2 adjusted for age and energy intake, the odds ratio of Model 2 was 2.773 (95% CI: 1.198–6.420) in the “≥UL” group compared with the “<EAR” group, and the risk of thyroid disease tended to be increased (*p* for trend = 0.023). In Model 3, adjusted for age, energy intake, weight status, exercise status, smoking status, and alcohol consumption, the odds ratio was 2.686 (95% CI: 1.161–6.215) in the “≥UL” group compared with the “<EAR” group, and the risk of thyroid disease tended to be increased (*p* for trend = 0.026). Also, in Model 4 adjusted for age, energy intake, weight status, exercise status, smoking status, alcohol consumption, breakfast, and frequency of eating out, the odds ratio was 2.554 (95% CI: 1.113–5.861) in the “≥UL” group compared with the “<EAR” group, and the risk of thyroid disease tended to be increased (*p* for trend = 0.34). Lastly, in Model 5, adjusted for age, energy intake, weight status, exercise status, smoking status, alcohol consumption, breakfast, frequency of eating out, education level, region of residence, household income level, and occupation, the odds ratio was 2.418 (95% CI: 1.010–5.787) in the “≥UL” group compared with the “<EAR” group, and the risk of thyroid disease tended to be increased (*p* for trend = 0.038). However, there was no risk of iodine intake incidence and thyroid disease in the male group and in the total subjects.

**Figure 6:**
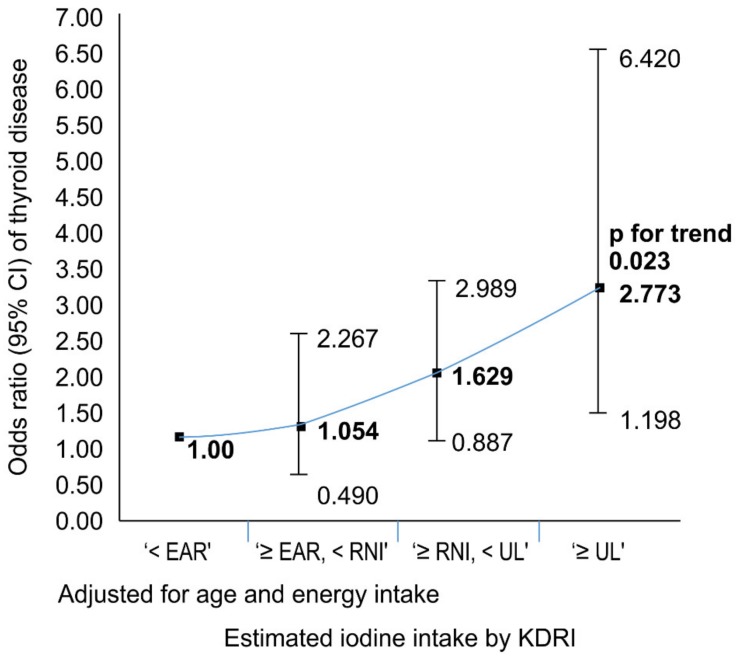
The odds ratio for thyroid disease by the estimated iodine intake in female.

This might support the idea that iodine intake by >UL (≥2400 µg/day) can increase the risk of thyroid disease in females (Figure 6). However, the same results were not observed in the male group. Also, dietary iodine intake did not indicate any risk of thyroid disease incidence.

## 4. Discussion

In this study, we investigated the relationship between iodine intake status and thyroid disease-related functions, and we found that as the estimated iodine intake increased, TSH increased and the risk of thyroid disease also increased. In Korea, iodine-replete areas generally have a high risk of thyroid disease and autoimmune thyroid disease tends to develop into hypothyroidism. Excessive iodine intake is considered to be an important factor in thyroid disease; however, the mechanism or cause is uncertain at present.

Currently, the iodine nutrition status is measured by dietary iodine intake or UIC. The dietary iodine intake is calculated by the iodine content of the food ingredient composition and measured using the 24 h dietary recall method, food record, and food frequency questionnaire. However, the 24 h dietary recall and food record methods do not accurately reflect the amount of iodine in soup stocks such as salt and kelp. In addition, the iodine content of the food composition varies greatly depending on the production area and production period. Also, the amount of iodine in the food is so low [3], and the dietary intake of iodine-rich foods also varies widely among individuals. Hence, it is very difficult to measure the amount of iodine in food and to obtain individual iodine intakes for meals [29,30].

Furthermore, iodine is mainly supplied by meals or drugs. The intake level is highly influenced by the environment, and iodine—which is a micronutrient—is present in a small amount in most foods and is concentrated in specific foods. It is difficult to accurately measure the iodine status [31,32]. Despite these limitations, the 24 h recall method is still valid for measuring dietary iodine intake in individuals. Hence, the UIC method is used more than the dietary iodine intake [8].

One of the most commonly used methods for measuring iodine nutrition status is the UIC as an epidemiologic criteria for the status of the population in the WHO [33]. Since, after metabolization in the body, more than 90% of iodine is excreted in the urine, UIC can estimate the recent intake of iodine by urinary iodine excretion amount and is used as a biomarker to determine the iodine status at the population level, which is a valuable and useful index [34,35]. However, UIC is inappropriate for evaluating the prevalence of iodine deficiency or excessive intake [16]. Therefore, in this study, we calculated and used the estimated iodine intake based on UIC.

Kim et al. [36] showed that 27 patients with Grave’s disease showed higher urinary iodine excretion than the normal group, and in a similar article [37], urinary iodine excretion was higher in patients with simple goiter (*n* = 17; 2.88 mg/L), hyperthyroidism (*n* = 42; 4.90 mg/L), hypothyroidism (*n* = 15; 4.57 mg/L), and thyroid cancer (*n* = 11; 6.18 mg/L) than in the normal group (2.11 mg/L). Additionally, in our study, the mean UIC based on prevalence was higher in the thyroid disease group than in the non-thyroid disease group, and the estimated iodine intake showed the same result, although the difference was not significant. These results indicate that UIC concentration is higher in the thyroid disease (such as goiter, hypothyroidism, and hyperthyroidism) group than in the disease-free group.

In this study, the correlation between UIC and dietary iodine intake showed a significant correlation with the total subject (r = 0.014, *p* < 0.001) and also in the female group (r = 0.089, *p* < 0.001), but, as mentioned, the male group showed the opposite result (r = −0.005, *p* < 0.001). Also, in another study [38], UIC was associated with dietary iodine intake (r = 0.60, *p* < 0.01), and as iodine intake increased, the UIC gradually increased, which indicates a positive correlation between iodine intake and UIC. Additionally, the correlation between the estimated iodine intake and dietary iodine intake also showed a significant correlation with the total subjects (r = 0.021, *p* < 0.001) and also in the female group (r = 0.095, *p* < 0.001), but the male group showed the opposite result (r = −0.001, *p* < 0.001). Comparing the dietary iodine intake and UIC between these groups and understanding the differences will enable us to determine the iodine nutrition status of the population more accurately.

In this study, the estimated iodine intake, which was calculated from UIC and dietary iodine intake, was divided into KDRI intervals to determine the risk of thyroid disease. These results show that the intake of iodine in Korea is higher than that in other countries, but there were more subjects who consume <95 ug (<EAR) and ≥2400 ug (UL) (Figure 2). Most subjects consumed >150 μg and <2400 μg, and 2.6% of them consume >2400 μg (dietary iodine intake) and 6.2% (estimated iodine intake) of iodine.

Furthermore, many other studies have shown that the median and upper limit of TSH increases with age [39,40,41]. In NHANES III, a higher TSH concentration was found to be associated with a higher UI/Cr excretion [42]. Additionally, TSH and FT4 levels were compared based on the presence of the disease. Moreover, similar results were found in the reference population: TSH levels were lower than that of the disease-free population. Therefore, a more in-depth study is required to clarify the link between TSH and iodine status; however, it has been confirmed that the excessive intake of iodine may be correlated with TSH.

In this study, the relationship between the estimated iodine intake, which was divided into KDRI intervals, and thyroid disease incidence was evaluated using the logistic regression analysis. It was found that the incidence of thyroid disease increased with increasing iodine intake. The correlation between the risk of thyroid disease according to the estimated iodine intake and the correlation between UIC and dietary iodine intake were significant only in the female group, but not in males.

These results support that the degree of response to iodine intake is higher due to the role of estrogen in women than in men, but more research is required on the concentration of estradiol and other cofactors [43,44,45,46,47].

One advantage of the study is that KNHANES VI (2013–2015) introduced TSH, FT4, TPOAb, and thyroid disease-related items, making it possible to observe the correlation of the iodine nutrition status with TSH and FT4 levels and to evaluate the effects of excessive iodine intake on thyroid disease. Additionally, the study cohort provided the most recently released nationally representative data of the Korean population.

However, there are some limitations to this study. First, KNHANES is a cross-sectional database; therefore, it cannot demonstrate the relationship between iodine intake and TSH or FT4 levels and thyroid disease, and it does not prove causality. Second, even though we have constructed a new iodine database for commercial foods in Korea, there is a limitation in the accurate determination of iodine intake by the food intake method. Finally, we did not consider various thyroid diseases.

The present study had limitations and further research is necessary to identify the factors contributing to the findings and to build accurate dietary sources. Despite these limitations and despite the need for further studies to identify the mechanisms involved in these findings and to build accurate dietary sources, this study provided important information. The study demonstrated the relationship between dietary iodine intake, UIC, and TSH along with a higher risk of thyroid disease-related hormone levels in groups with estimated iodine intake by over the UL (≥2400 µg/day).

## Figures and Tables

**Table 1:** Iodine nutrition status of the subjects.

	Total (*n* = 6095)		Unadjusted *p*-Value ^a^	Adjusted *p*-Value ^a,b^	Male (*n* = 2852)		Unadjusted *p*-Value	Adjusted *p*-Value	Female (*n* = 3243)		Unadjusted *p*-Value	Adjusted *p*-Value
	Non-Thyroid Disease (*n* = 5908)	Thyroid Disease (*n* = 187)			Non-Thyroid Disease (*n* = 2827)	Thyroid Disease (*n* = 25)			Non-Thyroid Disease (*n* = 3081)	Thyroid Disease (*n* = 162)		
	Mean ± SE	Mean ± SE			Mean ± SE	Mean ± SE			Mean ± SE	Mean ± SE		
Dietary Iodine (μg/day)	554.0 ± 35.8	458.8 ± 93.6	0.348	0.483	592.9 ± 60.0	533.2 ± 190.8	0.766	0.715	510.9 ± 34.3	445.8 ± 101.5	0.547	0.502
Estimated Iodine (μg/day)	780.0 ± 56.1	1108.1 ± 195.8	0.107	0.162	837.5 ± 97.8	775.7 ± 241.2	0.813	0.624	716.4 ± 39.0	1166.3 ± 226.2	0.051	0.067
UIC ^c^ (μg/L)	883.2 ± 92.1	1085.9 ± 183.9	0.322	0.400	913.5 ± 164.5	745.1 ± 235.8	0.558	0.462	849.8 ± 52.5	1145.5 ± 211.5	0.172	0.195

^a^*p*-value was calculated via the surveyreg procedure of SAS; ^b^ Adjusted for age and energy intake (energy intake was adjusted for age); ^c^ UIC = Urinary iodine concentration.

**Table 2:** UIC, TSH, free T4, and KDRI of the estimated iodine intake by sex.

Estimated Iodine Intake (µg/day)	Korean Dietary Reference Intakes (KDRI)				Unadjusted *p* for Trend ^d^	Adjusted *p* for Trend ^d,e^
	<EAR ^a^ (<95) (*n* = 850)	≥EAR, <RNI ^b^ (≥95, <150) (*n* = 987)	≥RNI, <UL ^c^ (≥150, 2400) (*n* = 3869)	≥UL (≥2400) (*n* = 389)		
	Mean ± SE	Mean ± SE	Mean ± SE	Mean ± SE		
**Total**						
Age	44.3 ± 0.8	44.4 ± 0.6	46.8 ± 0.3	50.7 ± 0.9	<.0001(+)	<.0001(+)
TSH (mIU/L)	2.5 ± 0.1	2.5 ± 0.1	2.8 ± 0.1	3.7 ± 0.4	0.008(+)	0.009(+)
UIC (μg/L)	111.1 ± 2.6	168.5 ± 3.4	632.6 ± 13.6	6903.2 ± 1202.3	<.0001(+)	<.0001(+)
FT4 (ng/dL)	1.3 ± 0.01	1.3 ± 0.01	1.2 ± 0.005	1.2 ± 0.01	<.0001(−)	<.0001(−)
**Male**						
Age	41.7 ± 1.1	42.8 ± 0.8	45.6 ± 0.3	50.1 ± 1.1	<.0001(+)	<.0001(+)
TSH (mIU/L)	2.1 ± 0.1	2.3 ± 0.1	2.6 ± 0.1	4.0 ± 0.7	0.024(+)	0.024(+)
UIC (μg/L)	109.8 ± 3.5	154.8 ± 3.9	580.0 ± 15.4	7404.0 ± 2222.9	0.002(+)	0.002(+)
FT4 (ng/dL)	1.3 ± 0.01	1.3 ± 0.0	1.3 ± 0.01	1.2 ± 0.02	<.0001(−)	0.002(−)
**Female**						
Age	46.0 ± 0.9	45.9 ± 0.8	48.3 ± 0.4	51.3 ± 1.3	0.002(+)	0.003(+)
TSH (mIU/L)	2.8 ± 0.1	2.8 ± 0.1	3.1 ± 0.1	3.4 ± 0.2	0.062(+)	0.079(+)
UIC (μg/L)	112.0 ± 3.3	181.6 ± 5.2	694.9 ± 21.6	6350.5 ± 597.7	<.0001(+)	<.0001(+)
FT4 (ng/dL)	1.2 ± 0.01	1.2 ± 0.01	1.2 ± 0.01	1.1 ± 0.01	0.0001(−)	0.001(−)

^a^ EAR: Estimated average requirement; ^b^ RNI: Recommended nutrient intake; ^c^ UL: Tolerable upper intake; ^d^ All p for trend were calculated by surveyreg procedure of SAS; ^e^ Adjusted for age and energy intake (age was adjusted for energy intake).

**Table 3:** Prevalence of thyroid disease, intake range, median, and mean according to the KDRI of the estimated iodine intake by sex.

Estimated Iodine Intake (µg/day)	Korean Dietary Reference Intake (KDRI) ^a^								
	<EAR^c^ (<95)		≥EAR, <RNI ^d^ (≥95, <150)		≥RNI, <UL ^e^ (≥150, 2400)		≥UL (≥2400)		Total
Total	*n* = 850		*n* = 987		*n* = 3869		*n* = 389		*n* = 6095
Intake Range	1.29–94.99		95.01–149.95		150.02–2399.36		2409.77–80672.0		1.29–80672.0
Median ± SE ^b^	72.0 ± 1.2		122.5 ± 1.1		362.5 ± 7.1		4102.9 ± 152.3		256.4 ± 5.5
Mean ± SE	69.4 ± 0.8		122.2 ± 0.6		558.0 ± 9.7		6301.4 ± 672.7		790.1 ± 54.7
**Prevalence of Thyroid Disease (*n*, weighted %)**									
Non-Thyroid Disease	830	97.2	964	97.6	3744	96.9	370	94.4	5908
Thyroid Disease	20	2.8	23	2.4	125	3.1	19	5.6	187
**Male**	*n* = 296		*n* = 453		*n* = 1910		*n* = 193		*n* = 2852
Intake Range	2.79–94.99		95.03–149.76		150.02–2391.91		2414.16–80672.0		2.79–80672.0
Median ± SE	74.6 ± 1.7		122.0 ± 1.4		355.7 ± 8.3		3823.3 ± 227.3		270.9 ± 6.5
Mean ± SE	71.7 ± 1.2		122.1 ± 0.9		543.5 ± 12.8		6772.6 ± 1228.9		836.9 ± 96.9
**Prevalence of Thyroid Disease (*n*, weighted %)**									
Non-Thyroid Disease	293	98.2	449	99.1	1894	99.2	191	99.3	2827
Thyroid Disease	3	1.8	4	0.9	16	0.8	2	0.7	25
**Female**	*n* = 554		*n* = 534		*n* = 1959		*n* = 196		*n* = 3243
Intake Range	1.29–94.97		95.01–149.95		150.29–2399.36		2409.77–36494.0		1.29–36494.0
Median ± SE	70.3 ± 1.6		122.7 ± 1.8		372.8 ± 12.7		4285.4 ± 189.5		235.4 ± 8.1
Mean ± SE	67.9 ± 1.0		122.3 ± 0.9		575.3 ± 13.7		5781.4 ± 399.6		740.7 ± 38.7
**Prevalence of thyroid disease (*n*, weighted %)**									
Non-Thyroid Disease	537	96.4	515	96.1	1850	94.2	179	89.0	3081
Thyroid Disease	17	3.6	19	3.9	109	5.8	17	11.0	162

^a^ KDRI group was classified based on the estimated iodine intake; ^b^ Standard error; ^c^ EAR: estimated average requirement; ^d^ RNI: Recommend nutrient intake; ^e^ UL: Tolerable upper intake.

**Table 4:** Logistic regression analysis of thyroid disease across KDRI of the estimated iodine intake by sex.

Estimated Iodine Intake (µg/day)	Korean Dietary Reference Intake (KDRI)				*p* for Trend ^d^
	<EAR ^a^ (<95)	≥EAR, <RNI ^b^ (≥95, <150)	≥RNI, <UL ^c^ (≥150, 2400)	≥UL (≥2400)	
**Total**					
Model 1	1 ^e^	0.832(0.409–1.692) ^f^	1.084(0.634–1.854)	1.788(0.820–3.898)	0.059(+)
Model 2	1	0.854(0.416–1.750)	1.092(0.631–1.891)	1.692(0.773–3.704)	0.096(+)
Model 3	1	0.887(0.428–1.838)	1.144(0.657–1.992)	1.796(0.815–3.960)	0.076(+)
Model 4	1	0.891(0.422–1.882)	1.166(0.662–2.056)	1.846(0.840–4.058)	0.066(+)
Model 5	1	0.847(0.390–1.836)	1.095(0.612–1.957)	1.726(0.760–3.923)	0.085(+)
**Male**					
Model 1	1	0.499(0.095–2.620)	0.440(0.108–1.792)	0.414(0.051–3.359)	0.720(−)
Model 2	1	0.474(0.089–2.528)	0.390(0.095–1.609)	0.325(0.041–2.568)	0.607(−)
Model 3	1	0.397(0.075–2.098)	0.317(0.073–1.378)	0.251(0.034–1.839)	0.549(−)
Model 4	1	0.405(0.080–2.054)	0.353(0.079–1.566)	0.278(0.036–2.161)	0.582(−)
Model 5	1	0.419(0.084–2.084)	0.311(0.070–1.378)	0.240(0.029–1.954)	0.5573(−)
**Female**					
Model 1	1	1.056(0.492–2.263)	1.667(0.908–3.061)	2.940(1.267–6.823)	0.014(+)
Model 2	1	1.054(0.490–2.267)	1.629(0.887–2.989)	2.773(1.198–6.420)	0.023(+)
Model 3	1	1.063(0.488–2.315)	1.608(0.873–2.961)	2.686(1.161–6.215)	0.026(+)
Model 4	1	1.048(0.479–2.292)	1.561(0.846–2.881)	2.554(1.113–5.861)	0.034(+)
Model 5	1	0.979(0.438–2.192)	1.426(0.766–2.654)	2.418(1.010–5.787)	0.038(+)

^a^ KDRI group was classified based on the estimated iodine intake; ^b^ Standard error; ^c^ EAR: Estimated average requirement; ^d^ *p* for trend was calculated by surveylogistic procedure of SAS; ^e^ Reference; ^f^ Odds ratio (95% CI, confidence interval); Model 1: Unadjusted model; Model 2: Adjusted for age and energy intake; Model 3: Adjusted for age, energy intake, weight status, exercise status, smoking status, and alcohol consumption; Model 4: Adjusted for age, energy intake, weight status, exercise status, smoking status, alcohol consumption, breakfast, and frequency of eating out; Model 5: Adjusted for age, energy intake, weight status, exercise status, smoking status, alcohol consumption, breakfast, frequency of eating out, education level, region of residence, household income level, and occupation.
